# Spatiotemporal dataset on moon jellyfish *Aurelia aurita* incidental observations in the Gulf of Riga and Eastern Gotland Basin, Baltic Sea

**DOI:** 10.1016/j.dib.2024.110880

**Published:** 2024-08-24

**Authors:** Astra Labuce, Laura Batare, Anda Ikauniece

**Affiliations:** Latvian Institute of Aquatic Ecology, Agency of Daugavpils University, 4 Voleru St., Riga LV-1007, Latvia

**Keywords:** Brackish, Cnidaria, Gelatinous, Scyphozoa, Zooplankton

## Abstract

This data article describes the occurrences of the moon jelly *Aurelia aurita* medusae in the Gulf of Riga and Eastern Gotland Basin (Baltic Sea) between 1998 and 2023. All data are incidental observations obtained during Latvian national monitoring cruises. Gelatinous zooplankton is not a standard group in regional marine monitoring, and jellyfish are not intentionally monitored within the framework of national marine monitoring by many countries across the Baltic Sea region. Hence, the information about *A. aurita* medusae occurrences in the area is scarce, especially long-term. The data also describe the seasonal dynamics of *A. aurita* medusae presence. Most frequently, observations occur from August to October in the studied area. However, the earliest observation of *A. aurita* medusae was recorded in July and the latest observation was in late November. These data provide relevant contributions to comprehending the seasonal and long-term dynamics of gelatinous zooplankton, specifically *A. aurita*. Moreover, the data can be used as a baseline for future studies on the eastern Baltic Sea addressing climate change and anthropogenic forcing impacts.

Specifications Table

**Table:** 

Subject	Marine Biology; Ecology
Specific subject area	Presence and absence data of jellyfish *Aurelia aurita* medusae in the brackish Gulf of Riga and Eastern Gotland Basin (Baltic Sea)
Type of data	Table (.csv format) Observations (presence/absence) are provided in a raw format; each data point includes information about the location and date-time of the observation.
Data collection	The environmental observations, including the presence of *A. aurita* medusae, were acquired during surveys concurrently with other routine monitoring parameters. If *A. aurita* medusae were visible in the surface water layers its presence was noted in the field sampling report. The presence of *A. aurita* was also reported if medusa was found in the mesozooplankton sample (collected following guidelines by the Baltic Marine Environment Protection Commission (HELCOM) [1], i.e., WP2 net, 100 µm mesh size, vertical tow). Data collection compiles sampling events between the years 1998 and 2023.
Data source location	Data are stored in the Latvian Institute of Aquatic Ecology, Agency of Daugavpils University, 4 Voleru St., Riga, LV-1007, Latvia. Data was obtained from the Gulf of Riga and the Eastern Gotland Basin (Baltic Sea); coordinates for every observation are provided in the raw dataset.
Data accessibility	Repository name: Zenodo Data identification number: 10.5281/zenodo.13124968 Direct URL to data: https://zenodo.org/records/13124968

## 1 Value of the Data

• Gelatinous zooplankton are not monitored within the framework of regular monitoring in the Baltic Sea, hence information about their occurrences in the region is scarce, especially on a long-term scale. This dataset contributes to filling this gap and can be useful for studies on comprehending the seasonal and long-term dynamics of *A. aurita* population in the Baltic Sea.
• This dataset offers valuable insights into the seasonal and geographical distribution patterns of *A. aurita* within the eastern Baltic Sea region.
• The data, when analysed in conjunction with environmental or socioeconomic data, can address the impact of climate change and anthropogenic forcing on *A. aurita* occurrences providing additional insights into the status of pelagic habitat in the region.
• Presence data supplement existing data compilations on gelatinous plankton and, inter alia, can be used to establish a baseline for future monitoring of gelatinous zooplankton, i.e., *A. aurita*, in the Baltic Sea.

## 2 Background

Climate change and various anthropogenic pressures, including overfishing, eutrophication, and oxygen depletion are expected to favour jellyfish populations [2]. Consequently, jellyfish may play a significant role in future coastal ecosystems. Despite this potential, the trophic ecology of jellyfish has historically been neglected [3]. In the Baltic Sea region, jellyfish presence, abundance and ecology are monitored and researched sporadically, using varying methodologies and approaches [[4], [5]]. Unfortunately, jellyfish monitoring lacks standardisation within the guidelines set by the Baltic Marine Environment Protection Commission (HELCOM) [1], which currently omit jellyfish despite their important trophic role, particularly *Aurelia aurita*, in the Baltic Sea ecosystem [4]. This lack of data hinders the incorporation of jellyfish population parameters into comprehensive environmental assessments and ecosystem-based solutions, perpetuating gaps in understanding their ecological impacts and responses to environmental changes. Therefore, there is a need for initiatives aimed at sharing data on jellyfish presence, abundance and ecology in the Baltic region [e.g., [[6], [7]]]. Such efforts not only enhance the scientific community's understanding of jellyfish population dynamics but also underscore the necessity of adopting standardised observation approaches to improve monitoring and conservation strategies.

## 3 Data Description

This data article presents an overview of the presence of moon jelly *Aurelia aurita* in the eastern Baltic Sea over 25 years, from 1998 to 2023 [8]. During this extensive timeframe, *A. aurita* medusae have been observed 217 times. In the years 2002 and 2019, *A. aurita* medusae were not observed in any of the sampling sites. Additionally, meteorological (e.g., wind and wave direction and height), hydrological (e.g., temperature and salinity), hydrochemical (nutrients) and other parameters (e.g. phytoplankton, chlorophyll a concentration) from the Latvian national monitoring program collected during the same visits as the *A. aurita* occurrences can be accessed on an online platform Latmare (https://latmare.lhei.lv/).

**Fig. 1:**
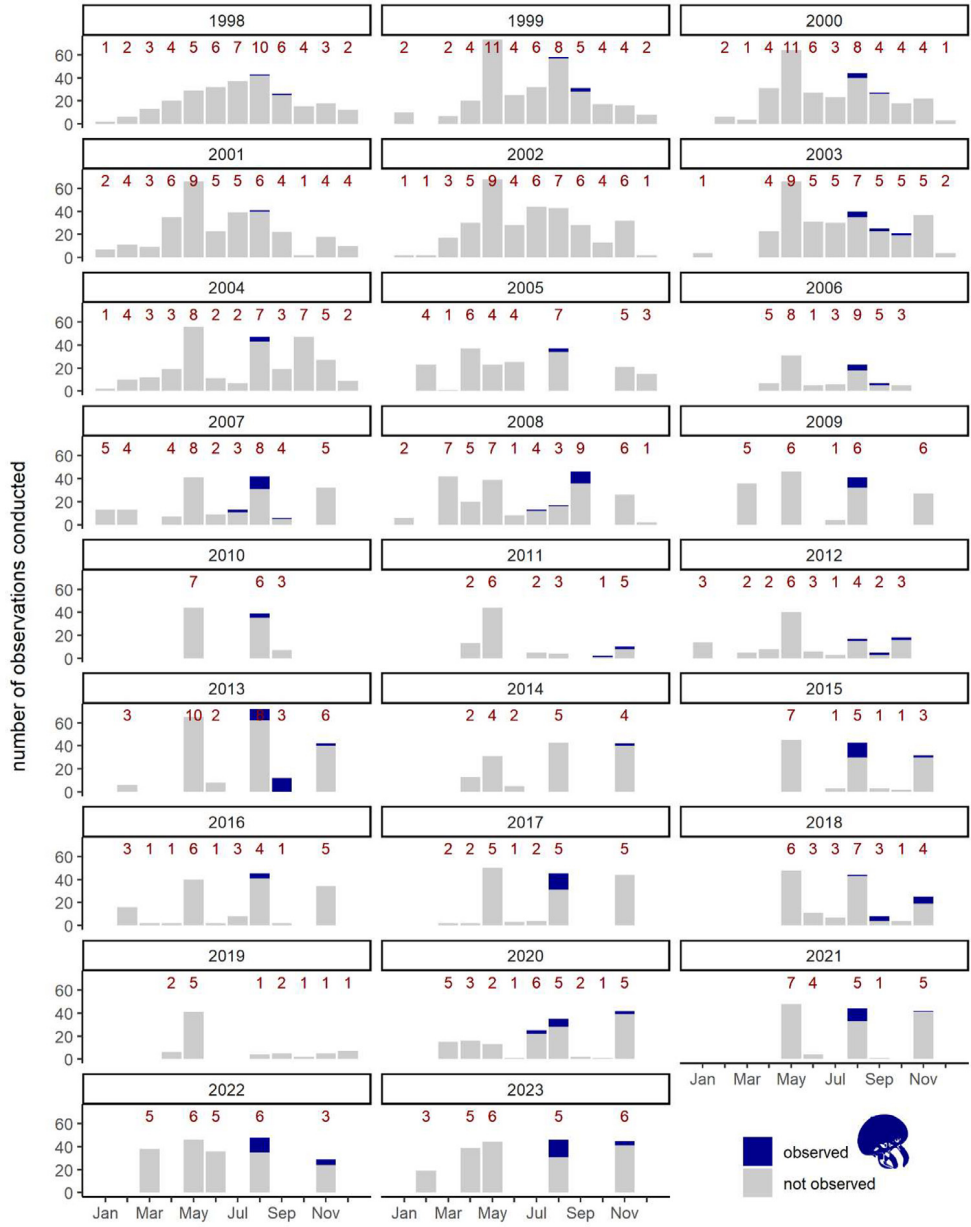
Absence and presence of *Aurelia aurita* medusae in the studied region. The Y-axis show the number of observations conducted each month. The number of observations can include visits to the same location if it is surveyed more times per respective month. Grey indicates a zooplankton sampling event without observation (absence) of *A. aurita* medusae; dark blue indicates detected presence of *A. aurita*; numbers on the columns represent the total number of days monitored during each respective month.

**Fig. 2:**
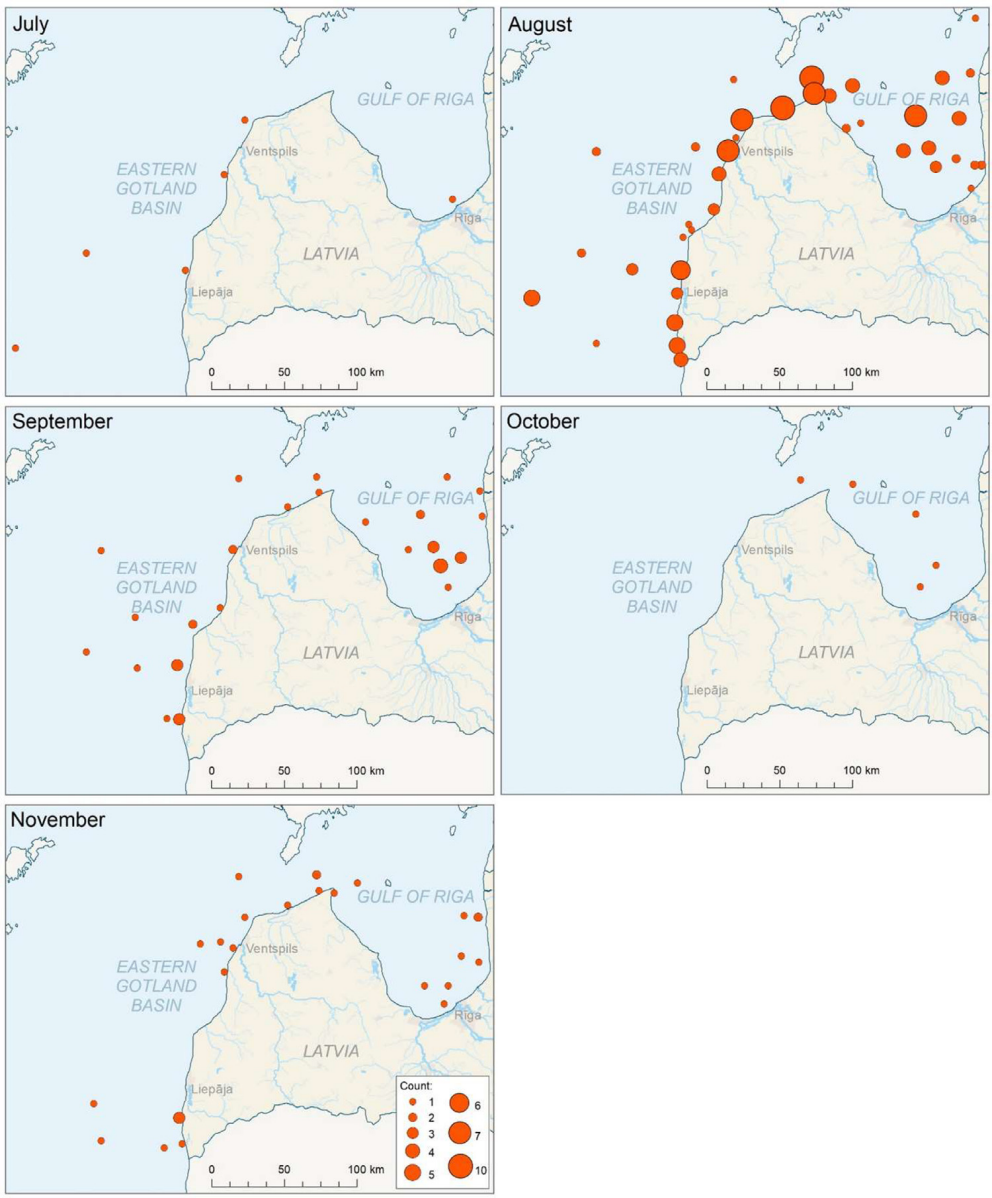
Monthly variations in the presence of *Aurelia aurita* medusae in the eastern Baltic Sea from 1998 to 2023. Circle size corresponds to the number of years *A. aurita* was observed at each location during the respective month (max. observations at one location in a month, i.e., August, is ten times during the studied period).

The raw data are visualized in Fig. 1, Fig. 2. Fig. 1 illustrates that *A. aurita* medusae are typically sighted from mid-July to November in the studied area. This seasonal pattern highlights the specific time window when the jellyfish are most prevalent. Whether medusae originate from local polyps or are transported from other regions of the Baltic Sea cannot be ascertained with available information, due to the lack of specific regional studies on *A. aurita* population ecology and genetics.

Fig. 2 presents the seasonal differences in the spatial distribution within the region. It shows that *A. aurita* medusae tend to appear earlier and in more locations within the waters of the Eastern Gotland basin, with numerous observations from July. In contrast, the presence of *A. aurelia* medusae in the Gulf of Riga during July has only been recorded once, specifically on 31 July 2020. Additionally, no other gelatinous zooplankton has been observed in the studied region and period.

## 4 Experimental Design, Materials and Methods

A designated researcher from a team conducting monitoring surveys observe the surrounding waters before collecting samples and records any significant findings. If *A. aurita* medusae are seen in the upper water layers at the sampling site, this observation is recorded in the field sampling protocol. According to the HELCOM monitoring guidelines [1], it is recommended to remove jellyfish from the WP2 zooplankton sampling net and repeat the sampling if jellyfish are detected. However, occasional instances occur where medusae are inadvertently included in zooplankton sample. These occurrences are typically noted during laboratory preparation of the zooplankton samples for analysis. Zooplankton samples were collected from the entire water column using a WP2 net (mesh: 100 µm mesh, diameter: 57 cm) by vertical tow at a speed approximately 0.5 m per second, following the monitoring guidelines set by HELCOM.

## Limitations

*A. aurita* medusae presence observations are primarily limited to daylight hours, though some have been made at night using vessel lights. In instances of high abundances, it is presumed that some medusae may be caught in a zooplankton net and subsequently recorded in the field sampling protocol, thereby reducing the likelihood of false negative data points when sampling is conducted during dark hours. The monitoring site visit time is not strictly predefined when zooplankton samples are collected from the entire water column; hence sampling is conducted upon arrival at the station to optimize cost-efficiency and reduce time spent at sea. However, the possibility of false negatives is higher in the autumn than in the summer due to the seasonal light cycle resulting in a longer dark hour period.

## Ethics Statement

The authors have read and follow the ethical requirements for publication in Data in Brief and confirm that the current work does not involve human subjects, animal experiments, or any data collected from social media platforms.

## CRediT authorship contribution statement

**Astra Labuce:** Conceptualization, Data curation, Resources, Visualization, Writing – original draft, Writing – review & editing. **Laura Batare:** Resources, Data curation. **Anda Ikauniece:** Resources, Writing – review & editing.

## Data Availability

Spatiotemporal Dataset on Moon Jellyfish (Aurelia aurita) Incidental Observations in the Gulf of Riga and Eastern Gotland Basin, Baltic Sea (Original data) (Zenodo).
